# Comparison of exercise training and repeated hot water immersion on vascular function in overweight older adults

**DOI:** 10.1113/EP094115

**Published:** 2026-07-19

**Authors:** Juliene G. Costa, Joao Carlos Locatelli, Kristanti W. Wigati, Louise H. Naylor, Helen Jones, Andrew Haynes, Daniel J. Green

**Affiliations:** ^1^ School of Human Sciences (Sports and Health Science) The University of Western Australia Crawley Western Australia; ^2^ Medical Physiology and Biochemistry Department, Faculty of Medicine Universitas Airlangga Surabaya Indonesia; ^3^ Research Institute for Sports and Exercise Science Liverpool John Moores University Liverpool UK; ^4^ Global Research Chair Manchester Metropolitan University Manchester UK

**Keywords:** aerobic exercise training, heat therapy, hot water immersion, obesity, vascular physiology

## Abstract

Recent studies suggest that heat exposure may benefit cardiovascular health, but in overweight and obese older adults, the chronic effects of heat therapy on artery function are still uncertain. We aimed to compare the effects of 12 weeks (3×/week, 40 min) of aerobic exercise (EXE) to hot water immersion (HWI) and an inactive control group (CON), on vascular function. Thirty‐one inactive older adults (58 ± 7years; 30.1 ± 3.8 kg/m^2^) were randomly assigned into three groups: EXE (cycle ergometry, *n* = 11), HWI (40°C, *n* = 11) or an inactive CON group (*n* = 9). Resting blood pressure (BP), pulse wave velocity (PWV; Sphygmocor), and flow‐mediated dilation (FMD%) and glyceryl trinitrate‐induced dilation (GTN%) in the brachial and femoral arteries (Doppler ultrasound) were assessed at baseline and after 12 weeks. The groups were matched for age, BMI and clinical parameters. For brachial FMD%, an interaction effect (*P* = 0.014) was observed, with *post hoc* analysis indicating that the increases observed in the EXE and HWI groups did not differ significantly, whereas the EXE group increased significantly more than CON. For femoral FMD%, an interaction effect (*P* = 0.047) indicated that the EXE group increased more than CON, with no difference between HWI and CON. Brachial and femoral GTN%, resting BP and PWV showed no main or interaction effect. These data suggest that 12 weeks of moderate aerobic exercise induces brachial and femoral artery endothelial function benefits relative to an inactive control group of older individuals with overweight and obesity, that brachial responses increased in response to both EXE and HWI, and the changes induced by exercise and hot water immersion did not statistically differ.

## Abstract (highlights)

- **What is the central question of this study?**
  Compared to exercise training, can repeated hot water immersion promote vascular changes in older individuals with overweight and obesity?
- **What is the main finding and its importance?**
  The findings of this study propose that hot water immersion represents a safe, feasible intervention to improve vascular function in individuals with overweight and obesity who may have reduced mobility and/or poor exercise adherence in traditional exercise programs. However, exercise is still fundamental for inducing broader vascular adaptations and global health improvements.

## 1 INTRODUCTION

Repeated passive heat exposure has been shown to be an effective therapeutic tool for reducing blood pressure (BP) and improving macrovascular function (Pizzey et al., 2021). In addition, observational evidence revealed reduced incidence of coronary heart disease and stroke in individuals who regularly undertake bouts of hot water immersion (HWI) (Ukai et al., 2020). Compared with air‐based heat therapies, HWI promotes faster and more efficient heat transfer to the body (Ramires et al., 1995), and also aids in venous return and cardiac filling due to the hydrostatic pressure effect of water immersion. This may result in more robust acute changes in core temperature, larger reductions in systemic vascular resistance and relatively preserved cardiac function (Atencio et al., 2025).

While the cardiovascular (CV) benefits associated with physical activity are well established (Tu et al., 2025), some individuals with medical conditions may have difficulty exercising and obtaining CV benefits due to the inability to engage in or perform activity at an intensity high enough to induce cardioprotective effects (Wen et al., 2025). This has encouraged the study of the effects of HWI and comparison to the effects of exercise interventions. The physiological response to thermal stress includes activation of thermoregulatory and CV mechanisms to promote heat dissipation via cutaneous vasodilation, with blood flow redistribution to the skin and increased peripheral arterial shear stress (Cramer et al., 2022). Arterial blood flow and shear stress changes induced by heat exposure are comparable to those seen during aerobic exercise (Brunt et al., 2016; Green et al., 2010, 2011, 2017), and shear stress has been postulated as a primary mechanism for improving macrovascular arterial function when repeated chronically (Green et al., 2010).

Recent studies have shown that repeated exposures to HWI had positive effects on CV parameters in young (Hoekstra et al., 2018) and healthy populations (Brunt et al., 2016; Carter, Spence, Atkinson, Pugh, Cable et al., 2014, Carter, Spence, Atkinson, Pugh, Naylor et al., 2014), compared with control groups. Few studies have analysed the chronic effects of HWI in different clinical populations. In heart failure patients (*n* = 16) (Oyama et al., 2013), a study reported improvements in inflammatory biomarkers and clinical symptoms after 2 weeks of HWI (40°C, 10 min). In individuals with type 2 diabetes (James et al., 2023), 8–10 HWI sessions (40°C) over 14 days induced a reduction in fasting insulin sensitivity and concentration. In women with polycystic ovary syndrome, improvements in femoral flow‐mediated dilatation (FMD) and cholesterol levels were reported after 8–10 weeks (Ely et al., 2019), while patients with coronary heart disease who underwent water‐based (34°C) circuit exercise training improved vascular endothelial function compared with land‐based exercise (Scheer et al., 2023). Taken together, these data suggest that the physiological effects of HWI may exert beneficial effects on physiological outcomes in high‐risk populations, but no studies have compared the impacts of exercise and HWI in older individuals with overweight and obesity.

The aim of the current study was therefore to compare the effects of a 12‐week aerobic exercise (EXE) intervention with HWI in older males and females with overweight and obesity. We assessed brachial and femoral artery function, alongside resting BP and arterial stiffness. We hypothesised that both interventions would elicit improvements after 12 weeks, compared with an inactive control group; and that the EXE and HWI interventions would elicit similar vascular changes.

## 2 METHODS

### 2.1 Participants

**FIGURE 1:**
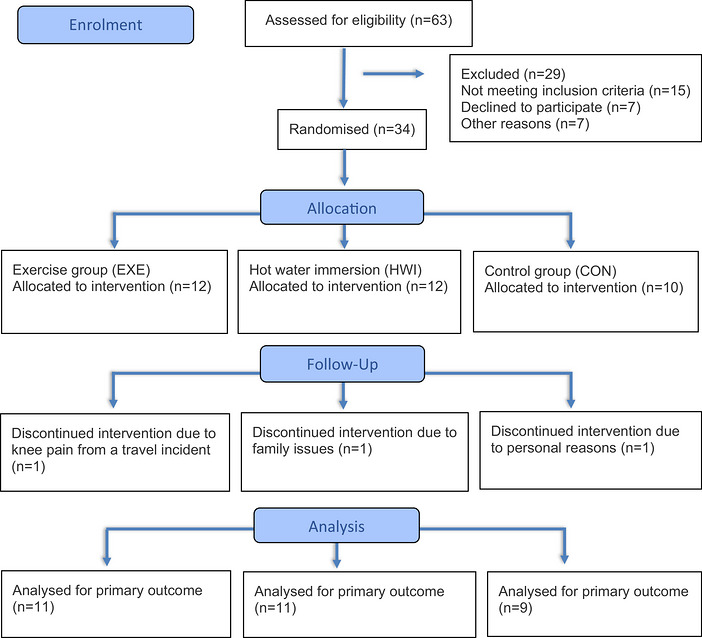
Flow diagram of the study.

The participants were recruited from the local community of Perth, Western Australia, through advertising. Potential participants initially completed an online screening questionnaire to determine suitability to attend a face‐to‐face appointment. At this appointment, the study details were explained, and they were free to ask any questions prior to signing informed consent. Further determination of eligibility occurred based on the following pre‐defined inclusion criteria: post‐menopausal women (more than 12 months without menstruation), between 45 and 70 years of age, or with a BMI of ≥30 kg/m^2^, or ≥27 kg/m^2^ with ≥1 weight‐related comorbidity (e.g. hypertension, dyslipidaemia or obstructive sleep apnoea), non‐smokers and relatively physically inactive (< 150 min per week of moderate intensity exercise) and no chronic experience with heat therapy in the last 6 months. Of the 63 individuals assessed for eligibility, 34 were enrolled in the study, and 31 completed the protocol (Figure 1). All participants provided written informed consent prior to participation in the study. This study conformed to the standards outlined in the *Declaration of Helsinki* and was reviewed and approved by The University of Western Australia Human Research Ethics Committee (2022/ET000738). This study is registered in the Australian New Zealand Clinical Trials Registry (ANZCTR) under the registration number CTRN12623000724673.

### 2.2 Study design

**FIGURE 2:**
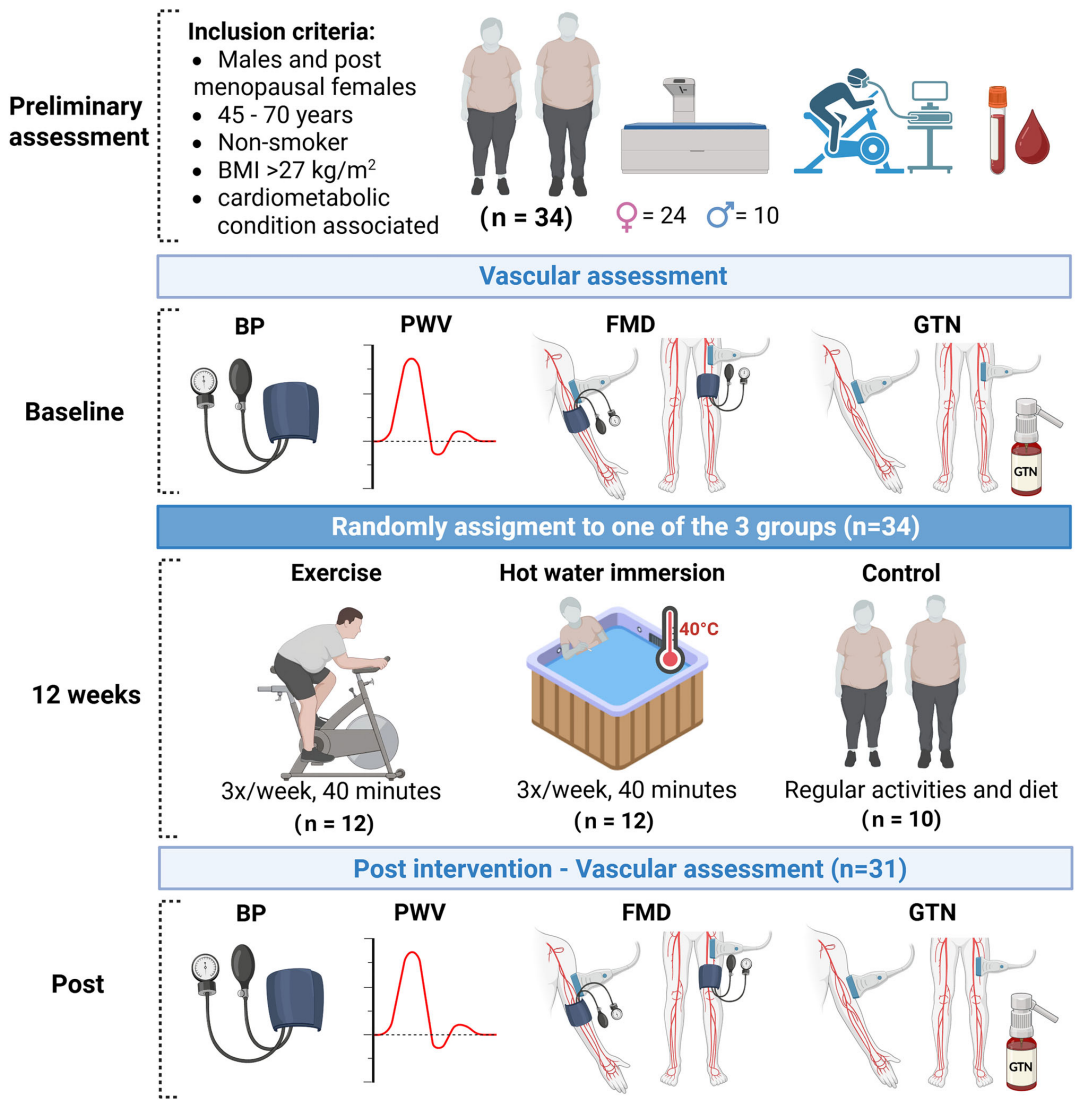
Study design.

In this randomised controlled trial, each participant attended one laboratory session for preliminary assessments (Figure 2) at the CV Research Laboratory at the University of Western Australia. Individuals were randomly assigned to one of three groups: exercise (EXE), HWI or a control (CON) group, to participate in a 12‐week intervention. An online tool (https://pickerwheel.com/) was used to generate a randomisation sequence, and block randomisation was employed to ensure similar numbers and sex‐matching across groups. All vascular outcomes (detailed below) were performed at baseline and at the end of the 12‐week period. The study took place from April 2024 to December 2024. This study was conducted and reported in accordance with the CONSORT guidelines (Hopewell et al., 2025).

### 2.3 Preliminary assessments

This session involved measuring height and body mass (used to calculate body mass index), body composition and aerobic capacity. Body composition was measured using dual‐energy X‐ray absorptiometry (Lunar iDXA, GE Healthcare Lunar, Madison, WI, USA), which derived indices including fat and lean mass, as well as visceral adipose tissue. Aerobic capacity (${\dot{V}}_{O_{2}\max}$) was assessed in response to continuous incremental cycling on a stationary bike ergometer. The test required participants to exercise at gradually increasing workloads, starting at 50 W and 60 rpm and increasing by 25 W every 2 min until volitional exhaustion. Heart rate (HR; Polar H10 HR monitor, Polar Electro Oy, Kempele, Finland) and rating of perceived exertion (RPE, 6–20 scale) were recorded in the last 15 s of each stage, and at the end of the test. Expiratory flow and gas composition were measured using a metabolic measurement system (Parvomedics TrueOne 2400, Salt Lake City, UT, USA). A pathology form was provided to each participant, instructing them to attend a specific pathology laboratory within 7 days. The participants were asked to fast for 8 h prior to receiving a blood sample for testing of fasting blood glucose, HbA1C and lipid levels.

### 2.4 Intervention groups

All participants were requested to maintain their usual exercise and dietary habits during the 12‐week intervention phase.

### 2.5 Aerobic exercise

For the EXE group, participants were required to attend the University of Western Australia (UWA) gym three times per week, for 12 weeks, to perform aerobic exercise on a stationary cycle ergometer. An Accredited Exercise Physiologist/Scientist individually prescribed the exercise programme based on the maximum heart rate (HR) achieved during the ${\dot{V}}_{O_{2}\max}$ test. The intensity and duration progressed as follows: familiarization during first 2 weeks (55–65% HR_max_, 30 min), week 3–6 (65–70% HR_max_, 40 min – being initial 5 min of warm up, 30 min in the zone, last 5 min cool down, maintaining 60–70 rpm), week 7–10 (70–75% HR_max_; 40 min, maintaining 60–70 rpm), week 11 and 12 (75–85% HR_max_, 40 min, maintaining 60–70 rpm). HR was monitored (Polar H10 HR monitor, Polar Electro Oy) by an exercise physiologist throughout all training sessions.

### 2.6 HWI

The HWI group was required to attend the University of Western Australia (UWA) School of Human Sciences (Exercise and Sport Science) three times per week for 12 weeks. The first 2 weeks were considered familiarisation, with sessions lasting 30 min at 40°C. The subsequent weeks (3–12 weeks) increased to 40‐min sessions at the same temperature. The participants were seated with water at the same level of the xiphoid process, with their arms submerged in an inflatable spa (San Francisco Smart Luxe HydroJet Pro 7, Bestway Inflatables & Material Corp., Shanghai, China). All sessions were monitored by an exercise physiologist.

### 2.7 Control group

Participants randomised to the control group were instructed not to start exercise and/or heat therapy sessions and were asked to maintain their usual exercise and dietary habits during the 12‐week intervention phase.

### 2.8 Outcome measures

All tests were performed in the morning, following at least an 8‐h fast and in abstinence from caffeine, and at least 24 h after strenuous physical activity (Muntner et al., 2019; Thijssen et al., 2019; Townsend et al., 2015). Participants rested quietly in a semi‐recumbent position for 15 min prior to any assessments. Post‐training assessments were conducted between 3 and 5 days after the final session to minimise acute exercise effects.

### 2.9 Resting BP

BP measurements were obtained using a *Dinamap* BP monitor (Patient Monitor V100, GE Healthcare, Chalfont Saint Giles, UK), with the participant in a semi‐recumbent position after a 20‐min period of quiet rest. Cuff size was selected based on a table of cuff sizes and arm circumferences to avoid artifacts arising from the selection of an inappropriate‐size cuff. A single investigator obtained all BP measurements (Muntner et al., 2019).

### 2.10 Arterial stiffness

Arterial stiffness was measured noninvasively (SphygmoCor® XCEL System (AtCor Medical Pty Ltd, Sydney, NSW, Australia) on the left side of the body. The carotid pulse was measured using a tonometer, while the femoral pulse was assessed through pulsations in a cuff placed around the thigh. Analysis of the waveform provides the following measurements: PWV (m/s; average of 2 consecutive readings) and Augmentation Index (AIx; %), the ratio of aortic augmented pressure to aortic pulse pressure (Townsend et al., 2015).

### 2.11 Flow‐mediated dilation

FMD was assessed in brachial and femoral arteries simultaneously by two trained sonographers, using 10 MHz linear‐array probes attached to a high‐resolution ultrasound machines (T3300, Terason, Burlington, MA, USA) in accordance with established guidelines (Thijssen et al., 2011, 2019). Participants were positioned supine and a pneumatic cuff connected to a rapid cuff inflator (AG 101, D.E. Hokanson, Inc., Bellevue, WA, USA) was placed around the left arm and left leg. On the left arm, the cuff was placed distal to the imaged artery, around the upper part of the left arm, and the brachial artery was scanned 2–5 cm above the antecubital fossa. On the left leg the cuff was placed ∼15 cm below the inguinal ligament and the superficial femoral artery was scanned in the proximal one‐third of the thigh, at least 5 cm distal from the bifurcation and above the cuff position. Images were collected using an insonation angle <60°, and baseline images were recorded for 1 min before the cuffs were inflated to 220 mmHg for 5 min. Recordings resumed 30 s before cuff deflation and continued for 5 min post‐deflation. Analysis of brachial and femoral artery blood flow and shear rate were performed using custom‐designed edge‐detection and wall‐tracking software, which is independent of investigator bias and has previously been comprehensively described (Woodman et al., 2001).

### 2.12 Endothelium‐independent glyceryl trinitrate‐mediated dilation

In accordance with current guidelines for vascular assessment (Thijssen et al., 2011, 2019), endothelium‐independent assessment was conducted at least 10 min after the FMD assessment to ensure a return to baseline diameter. Following image acquisition and optimisation of the brachial artery, a 1‐min baseline was recorded. A single sublingual dose of glyceryl trinitrate (400 µg) was then administered, and participants were instructed to refrain from swallowing within the first minute post administration to ensure absorption into the bloodstream. Ultrasound recordings in both arteries were then simultaneously collected by two trained sonographers, and these resumed 3 min after the glyceryl trinitrate spray and continued for an additional 5 min.

### 2.13 Data analysis

The sample size calculation was based on the study by Ely et al. (2019), who observed an increase in brachial FMD% after 8–10 weeks of water immersion compared to control (mean ± SD: 1.9  ±  2.5 vs. −0.1  ±  2.5%; effect size *f* = 0.40). Based on these, a minimum of 30 participants (*n* = 10 per group, accounting for 3 groups and 2 measures) were required to reject the null hypothesis at 95% power and α = 0.05. G*Power software (version 3.1.9.7) was used for calculating the sample size. Analysis of the Studentized residuals showed normality, as assessed by the Shapiro–Wilk test, and no outliers, as assessed by no Studentized residuals exceeding ±3 standard deviations. Data were expressed as means ± standard deviation. A one‐way ANOVA was used to compare the clinical parameters at baseline between the three groups. A two‐way mixed models ANOVA was performed to compare differences between EXE, HWI and CON at baseline and after the intervention for each of the following variables: resting BP, pulse wave velocity, brachial and femoral artery FMD, and glyceryl trinitrate‐induced dilation. An a priori decision was made to perform *post hoc* comparisons between paired data points using Bonferroni correction. For all comparisons, significance was set at *P* < 0.05. Statistical analyses were performed using SPSS Statistics version 29.0 (IBM Corp., Armonk, NY, USA).

## 3 RESULTS

Thirty‐one inactive older adults (58 ± 7years; BMI: 30.1 ± 3.8 kg/m^2^) were randomly assigned into EXE (*n* = 11), HWI (*n* = 11) or control group (*n* = 9). The clinical characteristics are presented in Table 1. The groups did not differ in age, weight, height, BMI, BSA, lipid or glucose levels; fat mass (kg) was the only variable that differed: the HWI group exhibited greater fat mass (kg) than the CON group. However, no differences were found between HWI and EXE, or between EXE and CON. In terms of medical conditions, 55% had high cholesterol, 19% had hypertension, 19% had thyroid disease, 19% had articular disease, 16% had reflux, 12% reported a mental health issue and 6% had asthma. The list of medications for each group is provided in Supporting information, Table S1. The baseline data for brachial and femoral diameters, velocity, flow and shear rate are provided in Table 2. No significant differences were observed between groups at baseline.

**TABLE 1:** Clinical characteristics at baseline.

	Overall (*n* = 31)	EXE (*n* = 11)	HWI (*n* = 11)	CON (*n* = 9)
Sample (males/females)	9 M/22F	3 M/8F	2 M/9F	4 M/5F
Age (years)	58 ± 7	57 ± 7	58 ± 6	58 ± 10
Weight (kg)	85.1 ± 11.9	87.5 ± 12.9	88.5 ± 12.7	78.0 ± 6.2
Height (m)	1.68 ± 0.08	1.69 ± 0.07	1.68 ± 0.09	1.67 ± 0.08
BMI (kg/m^2^)	30.1 ± 3.8	30.4 ± 2.8	31.4 ± 5.0	28.1 ± 2.2
BSA (m^2^)	1.9 ± 0.2	2.0 ± 0.2	2.0 ± 0.2	1.9 ± 0.1
Lean mass (kg)	47.1 ± 7.0	48.7 ± 7.9	46.4 ± 7.6	46.0 ± 5.1
Lean mass (%)	52.2 ± 6.7	56.1 ± 4.3	53.4 ± 8.8	59.6 ± 5.0
Fat mass (kg)	34.6 ± 9.3	35.4 ± 6.5	38.8 ± 12.0^#^	28.5 ± 4.8
Fat mass (%)	41.9 ± 7.1	42.0 ± 4.5	44.9 ± 9.4	38.2 ± 5.3
SBP (mmHg)	126 ± 11	129 ± 11	121 ± 11	129 ± 12
DBP (mmHg)	75 ± 10	77 ± 8	70 ± 11	78 ± 9
MAP (mmHg)	95 ± 9	98 ± 7	90 ± 10	98 ± 9
HR (bpm)	62 ± 11	63 ± 14	59 ± 11	63 ± 10
Cholesterol (mmol/L)	5.4 ± 0.8	5.6 ± 0.6	5.5 ± 0.9	5.5 ± 0.9
Triglycerides (mmol/L)	1.2 ± 0.5	1.3 ± 0.6	1.2 ± 0.4	1.1 ± 0.5
HDL (mmol/L)	1.6 ± 0.4	1.6 ± 0.4	1.7 ± 0.3	1.5 ± 0.3
LDL (mmol/L)	3.2 ± 0.7	3.4 ± 0.6	3.3 ± 0.9	3.0 ± 0.7
Glucose (mmol/L)	5.1 ± 0.5	5.1 ± 0.6	5.2 ± 0.4	5.1 ± 0.4
HbA1c (%)	5.3 ± 0.3	5.4 ± 0.3	5.2 ± 0.4	5.4 ± 0.2
HbA1c (mmol/L)	34.9 ± 3.8	36 ± 3.2	33.5 ± 4.8	35.4 ± 2.8
Medical conditions (medication)				
Thyroid diseases	6 (5)	2 (2)	2 (2)	2 (1)
Hypertension	6 (5)	2 (2)	2 (2)	2 (1)
High cholesterol	17 (2)	7 (1)	5 (0)	5 (1)
Reflux disease	5 (5)	1 (1)	4 (4)	0
Articular diseases	6 (1)	2 (0)	4 (1)	0
Mental health diseases (depression, anxiety)	4 (4)	2 (2)	2 (2)	0
Asthma	2 (1)	0	1 (0)	1 (1)

One‐way ANOVA.

^#^ HWI vs. control, *P *< 0.05. Abbreviations: BMI, body mass index; BSA, body surface area; CON, control group; DBP, diastolic blood pressure; EXE, exercise; HbA1c, glycated hemoglobin; HDL, high‐density lipoprotein; HR, heart rate; HWI, hot water immersion; LDL, low‐density lipoprotein; MAP, mean blood pressure; SBP, systolic blood pressure.

**TABLE 2:** Brachial and femoral arteries at baseline FMD and after 12 weeks.

	EXE (*n* = 11)		HWI (*n* = 11)		Control (*n* = 9)		*P* interaction	*P* time	*P* group
	BL	12 weeks	BL	12 weeks	BL	12 weeks			
**Brachial**									
BL diameter (mm)	3.50 ± 0.76	3.52 ± 0.77	3.33 ± 0.28	3.32 ± 0.43	3.48 ± 0.67	3.50 ± 0.63	0.996	0.717	0.742
BL velocity (cm/s)	9 ± 4	11 ± 4	10 ± 5	6 ± 1	10 ± 9	7 ± 3	0.287	0.318	0.287
BL flow (ml/min)	55 ± 38	68 ± 45	55 ± 29	33 ± 17	53 ± 11	43 ± 26	0.231	0.500	0.279
FMD response SRAUC	22056 ± 8072	21318 ± 8393	23336 ± 10235	21018 ± 9907	24504 ± 8887	24789 ± 8141	0.828	0.604	0.687
**Femoral**									
BL diameter (mm)	6.62 ± 1.47	6.59 ± 1.16	6.34 ± 0.87	6.16 ± 1.07	6.45 ± 0.77	6.52 ± 0.94	0.424	0.453	0.679
BL velocity (cm/s)	6 ± 3	5 ± 4	8 ± 5	6 ± 5	5 ± 2	4 ± 2	0.655	**0.015**	0.307
BL flow (ml/min)	138 ± 80	106 ± 88	144 ± 84	103 ± 87	96 ± 38	75 ± 32	0.675	**0.018**	0.417
FMD response SRAUC	9202 ± 5107	10990 ± 4810	9531 ± 3633	11813 ± 6402	11474 ± 6982	9906 ± 6029	0.410	0.261	0.952

*P*‐values shown in bold indicate statistical significance (*P* < 0.05). BL, baseline; FMD, flow‐mediated dilation; SRAUC, shear rate area under the curve.

### 3.1 Resting BP

**FIGURE 3:**
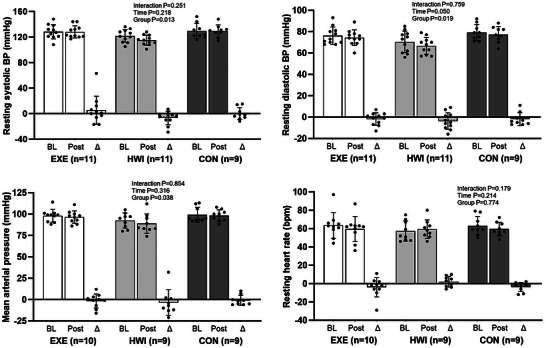
Differences between groups in resting systolic blood pressure, diastolic blood pressure, mean arterial pressure and heart rate. Two‐way mixed ANOVA. BP, blood pressure; BL, baseline; CON, control group; Δ, change: post minus baseline value; EXE, exercise; HWI, hot water immersion; Post, after 12 weeks assessment.

Systolic blood pressure showed a significant group effect (*P* = 0.013), but no time (*P* = 0.218) or interaction (0.251) effect (Figure 3). Diastolic blood pressure exhibited a group (*P* = 0.019), time (*P* = 0.050), but no interaction (*P* = 0.759) effect. Mean arterial pressure showed a group effect (*P* = 0.038), but no time effect (*P* = 0.316) or interaction effect (*P* = 0.854). The CON group showed a significant decrease from baseline (Δ: −4 ± 4 mmHg, *P* = 0.018), but the change did not differ from that in the HWI (*P* = 0.840) or EXE (*P* = 0.376) group. There was no time (*P* = 0.214), group (*P* = 0.774) or interaction (*P* = 0.179) effect on HR.

### 3.2 FMD

**FIGURE 4:**
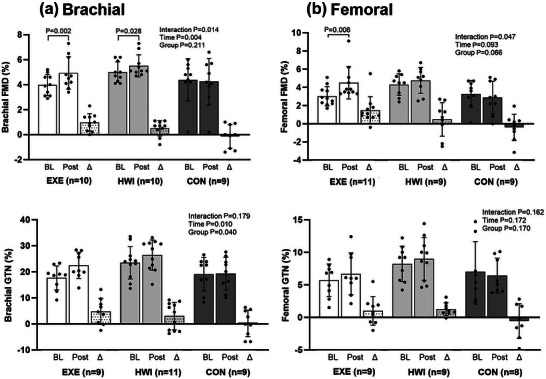
Differences between groups in brachial and femoral FMD% and GTN%. Two‐way mixed ANOVA. *P *< 0.05. BL, baseline; CON, control group; Δ, change: post minus baseline value; EXE, exercise; FMD, flow‐mediated dilation; GTN%, glyceryl trinitrate‐induced dilation; HWI, hot water immersion; Post, after 12 weeks assessment.

The brachial artery FMD% exhibited a significant interaction (*P* = 0.014) and time (*P* = 0.004) effect, but no group effect (*P* = 0.211) (Figure 4). The EXE (*P* = 0.002) and HWI (*P* = 0.028) groups had a significant increase from baseline, with no differences between these changes (ΔEXE: 1.0 ± 0.7 vs. ΔHWI: 0.5 ± 0.6%, *P* = 0.135). The CON group showed no difference compared to baseline (*P* = 0.674), and no difference in change compared to the HWI group (*P* = 0.093), but a significantly lower change compared to EXE (ΔEXE: 1.0 ± 0.7 vs. −0.1 ± 1.0%, *P* = 0.010).

The femoral artery FMD% showed a significant interaction (*P* = 0.047), but no group (*P* = 0.066) or time effect (*P* = 0.093). The EXE group showed a significant increase from baseline (*P* = 0.008), whereas the HWI (*P* = 0.460) and CON (*P* = 0.431) groups showed no change from baseline. A larger change in EXE was apparent when compared to the CON group (ΔEXE:1.5 ± 1.5 vs. ΔCON:−0.4 ± 1.4%, *P* = 0.011), with no difference compared to the HWI group (ΔHWI:0.5 ± 1.9, *P* = 0.198).

### 3.3 Glyceryl trinitrate‐mediated dilation

Brachial glyceryl trinitrate‐induced dilation (GTN%) showed a time effect (*P* = 0.010) and a group effect (*P* = 0.040), but no interaction effect (*P* = 0.179) (Figure 4). There was no significant interaction (*P* = 0.162), time (*P* = 0.172) or group effect (*P* = 0.170) in femoral GTN%.

### 3.4 Arterial stiffness

**FIGURE 5:**
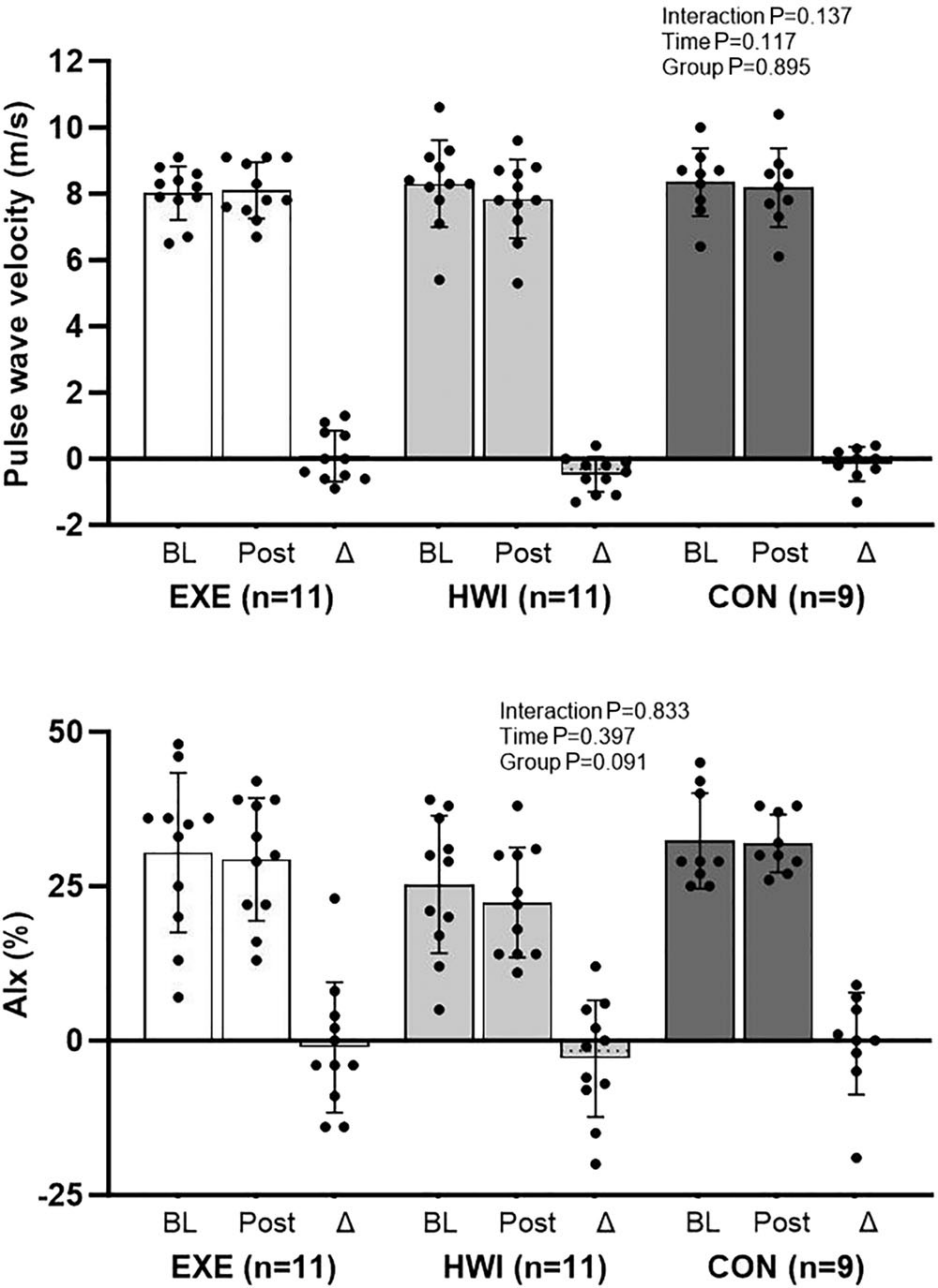
Differences between groups in pulse wave velocity and AIx. Two‐way mixed ANOVA. AIx, augmentation index; BL, baseline; CON, control group; Δ: change: post minus baseline value; EXE, exercise; HWI, hot water immersion; Post, after 12 weeks assessment.

In terms of PWV, no time (*P* = 0.117), group (*P* = 0.895), or interaction effect (*P* = 0.137) was evident (Figure 5).

## 4 DISCUSSION

This is the first study, to our knowledge, to compare the effects of HWI versus supervised EXE in overweight older participants. The main findings of the present study were that EXE and HWI increased brachial FMD%, and that EXE increased femoral FMD%. In our study, the pattern of change we observed in FMD% responses across the EXE, HWI and CON groups was consistent in both the brachial and femoral arteries.

The EXE and HWI groups exhibited significant increases in brachial FMD% after 12 weeks of intervention, with *post hoc* analysis showing that the magnitude of increase did not differ between these groups, while the EXE change was significantly larger than that in CON. The EXE and HWI groups also exhibited increases in femoral FMD%, but only the EXE group differed significantly from baseline. In keeping with the brachial data, the femoral FMD% EXE‐induced change was significantly larger than that in CON, whereas the EXE and HWI changes did not statistically differ. These results suggest that exercise induces a systemic vascular adaptation and that the lack of femoral adaptation with HWI may reflect an insufficient stimulus magnitude to promote adaptations comparable to those induced by exercise. In this study, the shear rate area under the curve response to our FMD stimulus did not significantly differ before or after any of the interventions (Table 2) for either artery, indicating that the stimulus magnitude cannot account for the changes we observed in FMD per se. The exercise intervention may elicit more systemic mechanisms than HWI (Cullen et al., 2020); evidence suggests that exercise, more than heat therapy, stimulates circulating factors such as heat shock proteins and vascular endothelial growth factor, which, together with nitric oxide, are essential for vascular adaptation (Morton et al., 2007).

Our FMD% results are broadly consistent with the findings of Bailey and colleagues (Bailey et al., 2016), who demonstrated that 8 weeks (3×/week, 30 min per session) of HWI (at 42°C) induced similar adaptations in brachial FMD% to moderate intensity cycling (70% HR_max_) in healthy young females (25 ± 5 years, *n* = 18). In another study (Akerman et al., 2019) of participants with peripheral artery disease (PAD, 74 ± 10 years; 28.5 ± 10.1 kg/m^2^, *n* = 22), no changes in brachial FMD% were observed after 12 weeks of EXE (2×/week, 30 min of walking + 60 min of circuit exercises) or HWI (at ∼39°C, 3–5 days/week, +15–30 min of callisthenics exercises). The lack of changes observed in that study may, in part, be explained by the interventions used or idiosyncratic responses in a PAD population. In older adults with overweight or obesity, a meta‐analysis of 36 studies (*n* = 1418) demonstrated that aerobic exercise, when performed at least three times per week, enhances FMD in individuals with and without CV disease (Li et al., 2023). Studies have also shown that shear stress in inactive muscle groups (e.g. the upper limbs during lower limb exercise) is exercise and intensity‐dependent (Montalvo et al., 2022; Thijssen et al., 2009), suggesting that cycling induces a greater increase in shear rate than walking combined with resistance training. In our study, brachial FMD% responses to HWI and cycle EXE were both significant. Similarly, previous studies have shown improvements in brachial FMD% after increases in shear induced by cycle exercise (Green et al., 2010), as well as passive forearm (Carter et al., 2013; Naylor et al., 2011) and the heating of the lower limbs (Carter et al., 2014b). In each of those experiments, truncation of shear increase using partial cuff inflation on one upper limb abolished the brachial FMD% adaptation. These studies therefore demonstrated that shear rate plays an important role in inducing adaptation in conduit arteries in humans (Green et al., 2017).

In our study, we did not find changes in brachial or femoral GTN% after exercise and HWI. Because GTN responses reflect vascular smooth muscle sensitivity, the absence of change suggests that the adaptations we observed were not driven by alterations in smooth muscle responsiveness. In accordance with our results, Brunt et al. (2016) performed 8 weeks of HWI (40.5°C, 4–5/week, *n* = 10), versus thermoneutral water immersion (sham, *n* = 10), in young healthy participants and did not show improvement in brachial GTN%, despite improvement in brachial FMD%. The increases in FMD we observed are consistent with improvements in endothelium‐dependent vasodilation.

In the present study, we did not observe any changes in arterial stiffness when comparing CON, EXE or HWI. Consistent with this, a study (Kaiser et al., 2025) of adults with untreated hypertension (30–60 years) showed no arterial stiffness improvements after 8–10 weeks (3/4× times per week) of aerobic exercise (cycle ergometer for 40 min, *n* = 20) or water immersion (40°C, 45 min, *n* = 21). Artery wall stiffness measured via PWV is commonly classified as central when analysed by the femoral–carotid PWV and peripheral when analysed by the brachial–ankle PWV index. Ashor et al. (2014) demonstrated in a meta‐analysis that greater reductions in response to aerobic exercise are observed in participants with stiffer arteries (PWV > 8 m/s), and as a result of higher‐intensity interventions lasting more than 10 weeks. The improvement seems to be more pronounced in the peripheral conduit arteries (assessed by brachial–ankle PWV), compared to the central arteries (assessed by femoral–carotid PWV) (Green et al., 2004; Rakobowchuk et al., 2008). Another meta‐analysis studied older participants with obesity (Montero et al., 2014), and demonstrated no improvements in brachial–ankle PWV or carotid–femoral PWV in response to aerobic exercise. The authors speculated that weight‐bearing exercise may hinder improvements in arterial stiffness in obese individuals, as carrying extra weight may lead to intense muscle contractions and higher intramuscular pressures. Other factors limiting adaptation of PWV to EXE and HWI interventions may include heightened sympathetic activity and/or baroreflex impairment in obesity (Montero et al., 2013).

Lower‐limb conduit arteries may be less adaptable or slower to respond to interventions than upper‐limb arteries, and may require a stronger stimulus to achieve lasting adaptation. In our study, the EXE progression may be responsible for the larger adaptation observed than HWI Unlike exercise, HWI does not allow progressive overload through intensity modulation. Future research should explore HWI programmes with different durations or frequencies that could be a sufficient physiological stimulus to promote femoral artery adaptation for individuals with overweight or obesity.

The findings from this study indicate that HWI, similar to exercise, is characterised by low rates of adverse events and high adherence. When conducted three times per week with 40‐min sessions over a 12‐week period, HWI has shown potential for improving vascular health in older adults with overweight or obesity. Based on these results, it is recommended that health practitioners consider incorporating HWI as a viable intervention for this population. Additionally, promoting HWI as an accessible form of exercise could enhance engagement and adherence, and progression to exercise interventions as tolerance and function improve. Furthermore, community‐based initiatives may increase awareness and participation in HWI, thereby fostering healthier lifestyles among older adults at risk of CV disease.

The present study has some limitations. We progressively increased exercise intensity (%HR_max_) and session duration throughout the EXE intervention, whereas the HWI group only extended immersion session duration over 12 weeks. It is also possible that distinct forms of training or water immersion protocols might produce different results. Our choice of each intervention was informed by ecological validity; we compared EXE and HWI interventions that represented similar durations, intensity and heat exposure to those broadly adopted in community settings.

Our sample size per group limited study power, but we still observed statistical differences in some variables and our study was of similar sample size to many previous experiments (Akerman et al., 2019; Bailey et al., 2016; Brunt et al., 2016; Ely et al., 2019). Finally, although no adverse events were observed in our study, the risk of postural hypotension and falls is relevant in older populations. We recommend supervised interventions with CPR trained staff in all higher risk and clinical populations.

### 4.1 Conclusions

These data suggest that 12 weeks of moderate aerobic exercise induces brachial and femoral artery endothelial function benefits relative to an inactive control group of older individuals with overweight and obesity, that brachial responses increased in response to both EXE and HWI, and the changes induced by exercise and HWI did not statistically differ.

Our findings should encourage further investigation of the possible beneficial impacts of HWI as a therapy for individuals who may, initially at least, find participation in structured exercise programmes difficult to achieve.

## AUTHOR CONTRIBUTIONS

Juliene G. Costa: acquisition, analysis and interpretation of data for the work, writing—original draft. Joao Carlos Locatelli and Kristanti W. Wigati: acquisition, methodology, analysis and interpretation of data for the work; data curation; revised the manuscript critically for important intellectual content. Helen Jones, Andrew Haynes and Louise H. Naylor: analysis and interpretation of data for the work; data curation; revised the manuscript critically for important intellectual content. Daniel J. Green: conceptualisation, supervision, data analysis, analysis and interpretation of data for the work and revised the manuscript critically for important intellectual content. All authors approved the final version of the manuscript; agreed to be accountable for all aspects of the work in ensuring that questions related to the accuracy or integrity of any part of the work are appropriately investigated and resolved; and all persons designated as authors qualify for authorship, and all those who qualify for authorship are listed.

## CONFLICT OF INTEREST

None declared.

## GENERATIVE AI STATEMENT

The authors confirm that no artificial intelligence tools, including large language models (LLMs), were used in the drafting or revision of this manuscript. All content was conceived, written and approved solely by the authors.

## Supporting information



Table S1. List of medical conditions and medications per group.

## Data Availability

The source data are available to verified researchers upon request by contacting the corresponding author.
