# Supplementary material for: Comparison of exercise training and repeated hot water immersion on vascular function in overweight older adults
Source: Exp Physiol. 2026 Jul 19:10.1113/EP094115. Online ahead of print. doi: 10.1113/EP094115 (PMC13394765; doi:10.1113/EP094115)
Supplement: Supplementary file 1 — Table S1. List of medical conditions and medications per group. [file EPH-9999-0-s001.docx]

**Supplementary Table 1.** List of medical conditions and medications per group.

| **Group** | **Medical condition (number of participants with a diagnosis)** | **N of participants taking medication** | **Medication** |
| --- | --- | --- | --- |
| EXE | Thyroid diseases (n=2) | 2 | Thyroxine  Thyroxine |
|  | Hypertension (n=2) | 2 | Irbesartan + amlodipine  Irbesartan |
|  | High cholesterol (n=2) | 1 | Rosuvastatin |
|  | Reflux disease (n=1) | 1 | Pariet |
|  | Articular diseases (n=2) | 0 | - |
|  | Mental health conditions (2)  (anxiety, depression) | 2 | Fluoxetine  Lexapro |
|  | Asthma (n=0) | 0 | - |
| **Group** | **Medical condition (number of participants with a diagnosis)** | **N of participants taking medication** | **Medication** |
| HWI | Thyroid diseases (n=2) | 2 | Thyroxine  Thyroxine |
|  | Hypertension (n=2) | 2 | Irbesartan  Irbesartan |
|  | High cholesterol (n=5) | 0 | - |
|  | Reflux disease (n=4) | 4 | Omeprazole  Omeprazole  Nexium  Salpraz |
|  | Articular diseases (n=4) | 1 | Voltaren |
|  | Mental health conditions (2)  (anxiety, depression) | 2 | Fluoxetine  Citalopram |
|  | Asthma (n=1) | 0 | - |
| **Group** | **Medical condition (number of participants with a diagnosis)** | **N of participants taking medication** | **Medication name** |
| EXE | Thyroid diseases (n=2) | 1 | Thyroxine |
|  | Hypertension (n=2) | 1 | Amlodipine |
|  | High cholesterol (n=5) | 1 | Atorvastatin |
|  | Reflux disease (n=0) | 0 | - |
|  | Articular diseases (n=0) | 0 | - |
|  | Mental health conditions (0) | 0 | - |
|  | Asthma (n=1) | 1 | Symbicort |
